# Diagnosis of Pulmonary Embolism in Unenhanced Dual Energy CT Using an Electron Density Image

**DOI:** 10.3390/diagnostics11101841

**Published:** 2021-10-05

**Authors:** Kyungsoo Bae, Kyung-Nyeo Jeon

**Affiliations:** 1 Department of Radiology, Institute of Health Sciences, School of Medicine, Gyeongsang National University, Jinju 52727, Korea; ksbae@gnu.ac.kr; 2 Department of Radiology, Gyeongsang National University Changwon Hospital, Changwon 51472, Korea

**Keywords:** dual-energy computed tomography, pulmonary embolism, electron density, contrast media, computed tomography pulmonary angiography

## Abstract

Dual-energy computed tomography (CT) is a promising tool, providing both anatomical information and material properties. Using spectral information such as iodine mapping and virtual monoenergetic reconstruction, dual-energy CT showed added value over pulmonary CT angiography in the diagnosis of pulmonary embolism. However, the role of non-contrast-enhanced dual energy CT in pulmonary embolism has never been reported. Here, we report a case of acute pulmonary embolism detected on an electron density image from an unenhanced dual-energy CT using a dual-layer detector system.

## 1. Introduction

Acute pulmonary embolism (PE) is a potentially fatal medical condition if its diagnosis is delayed. Computed tomography (CT) pulmonary angiography (CTPA) is the gold standard in the diagnosis of PE. Dual-energy CT (DECT) is a promising tool providing both anatomical information and material properties. An iodine map from DECT shows added value over CTPA in the diagnosis of PE [1]. In CT with suboptimal enhancement, monoenergetic image reconstruction can improve the diagnostic accuracy of PE by increasing vascular attenuation and the contrast-to-noise ratio [2]. However, in some patients, without clinical suspicion of PE, CT scans can be performed without intravenous contrast administration. The role of an unenhanced DECT in detecting PE has not been reported yet. Here, we report a case of acute PE detected on an electron density (ED) image from an unenhanced DECT obtained using a dual-layer detector system.

## 2. Case Report

**Figure 1:**
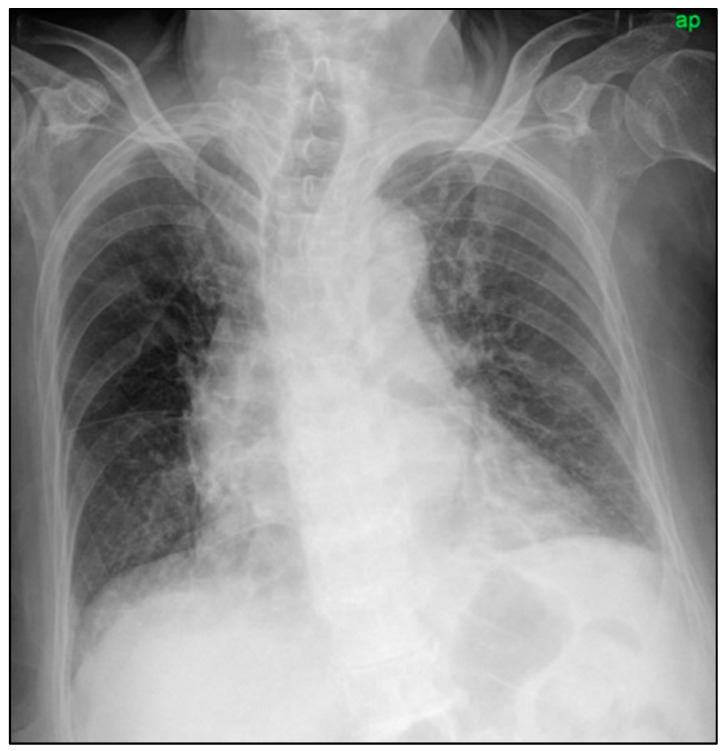
Supine anteroposterior chest radiograph showing suspicious infiltrates in the right upper lobe and both lower lobes.

This study was approved by the Institutional Review Board of our hospital. A 90-year-old woman was transferred to the emergency department with a cough, sputum, and dyspnea on exertion for a week. She was on medication for hypertension. Her white blood cell count was 12.33 × 10^3^ μL and her erythrocyte sedimentation rate was 37 mm/h. Reverse transcription polymerase chain reaction (RT-PCR) test for SARS-CoV-2 was negative. Her serum creatinine level was 1.17 mg/dL. Her estimated glomerular filtration rate (eGFR) was 48 mL/min/1.73 m^2^. A chest radiograph showed subtle increased opacities in the right upper lobe and both lower lobes (Figure 1).

**Figure 2:**
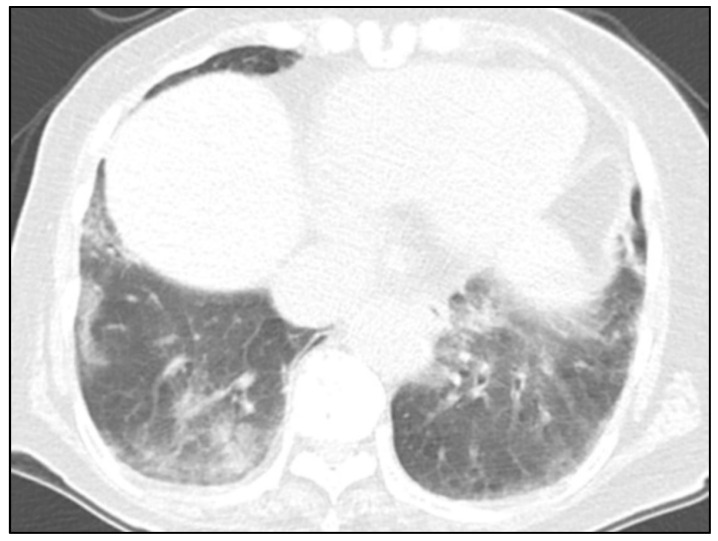
Chest CT image in lung window setting showing patchy consolidation and ground-glass opacities in both lower lobes.

Since pneumonia was suspected, a chest CT scan was performed without contrast enhancement. CT was obtained using a dual-layer detector spectral CT scanner (IQon, Philips Healthcare, Best, The Netherlands). The scanning parameters were as follows: 120 kVp, 140–250 mA (reference mAs, 73), pitch of 0.609, rotation time of 0.4 s, 64 × 0.625 mm collimation, 1 mm slice thickness, 1 mm slice increment, and smooth filter (filter A). Chest CT images showed patchy areas of consolidation and ground-glass opacities in both lower lobes, suggestive of pneumonia (Figure 2). There was a focal fibrotic scar in the right upper lobe. The patient was treated with antibiotics. Chest radiographs showed gradual improvement. However, the patient complained of persistence of dyspnea. Blood oxygen saturation level was 85% at room air and 99% after supplement of oxygen via nasal prongs at 3 L/min.

**Figure 3:**
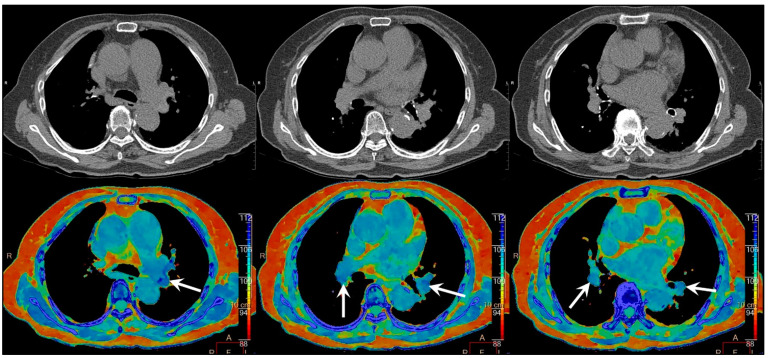
Upper row: unenhanced chest CT images in mediastinal window setting showing unremarkable findings. Lower row: color overlayed electron density images showing multiple high-density ED areas in both pulmonary arteries (arrows). Scale bar is shown on the right. Materials with higher electron densities are shown in dark blue. Lower-density materials are shown in light blue or yellow. ED, electron density.

**Figure 4:**
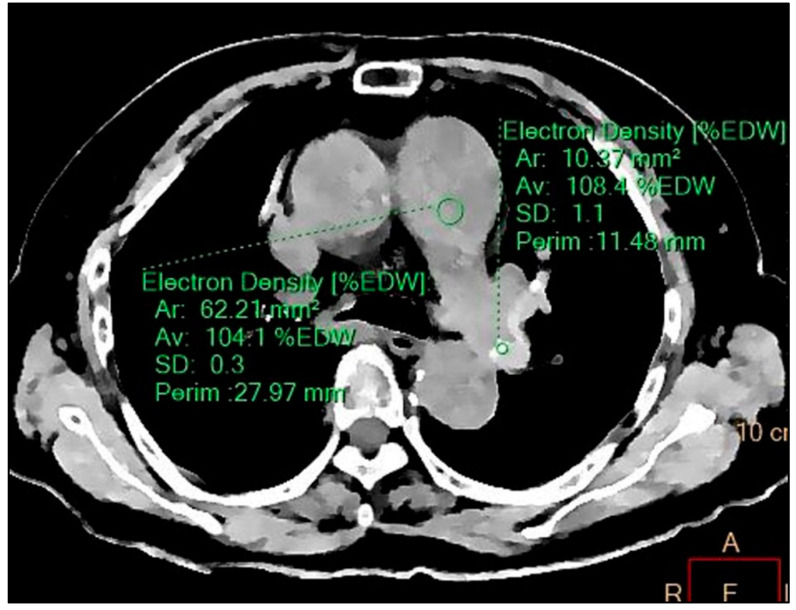
Mean electron ED values measured using circular region of interest on ED image: 108.4 (%EDW, percentage relative to the ED of water) in the high-density area and 104.1 in other areas of the pulmonary artery. ED, electron density.

**Figure 5:**
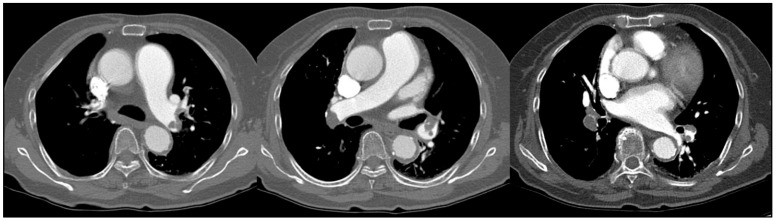
CT pulmonary angiography shows multiple PEs. Locations of PEs correspond to areas of dark blue areas on color overlayed ED images in Figure 3. PE, pulmonary embolism; ED, electron density.

A review of the unenhanced chest CT images revealed unremarkable findings except pneumonia. ED images were retrospectively reconstructed from a spectral based image of DECT. ED images showed multifocal areas of high ED in both interlobar, the left upper lobe, and both lower lobe pulmonary arteries (Figure 3). The ED value measured at a high-density area was 108 %EDW, while the ED value of pulmonary artery without a high density was 104 %EDW (Figure 4). Serum D-dimer level was 10.46 μg/dL. With a suspicion of PE, CTPA was performed on the third day of admission. CTPA showed multiple thrombi in pulmonary arteries. The locations of thrombi corresponded to areas of high ED on ED images (Figure 5).

CT venography revealed deep vein thrombosis in left distal popliteal and calf veins. The patient received anticoagulation therapy. Follow-up CTPA obtained 12 weeks later showed complete resolution of PE.

## 3. Discussion

Although there are several original research studies or case series on the detection of PE using signs of a hyperdense lumen in non-contrast-enhanced CT, density differences between intravascular blood and thrombus are usually not sufficiently big enough to be noticed without a contrast enhancement [3,4,5,6,7]. Protein content in red blood cells rather than fibrin in thrombus has been shown to be related to hyperdensity in CT [8]. Therefore, the visualization of PE on an unenhanced CT depends on the thrombus age and the patient’s hematocrit level at the time of imaging [5]. According to a case control study by Sun et al. [9], non-contrast CT showed a high negative predictive value (>90%) for central PE identification. However, it was neither sensitive nor specific enough to accurately detect central PE.

Conventional CT images depict the amounts of total beam attenuation in each voxel of body tissue when an X-ray passes through the body. Total X-ray attenuation is a result of two physical interactions between X-ray photons and the body, including the photoelectric absorption and Compton scattering known to depend on X-ray energy, effective atomic number, and ED of the tissue. By measuring X-ray attenuation at two different energies, DECT allows the estimation of the effective atomic number and ED of each voxel. DECT can produce more accurate ED values than a conventional CT [10,11,12]. The ED value derived from DECT is expressed as a percentage relative to the ED of water (%EDW). Commercially available DECT systems are classified into two main categories: source-based (dual rotation, dual source, rapid kilovoltage switching) and detector-based (dual-layer) systems [13]. In a dual-layer detector CT system, as used in the preset study, spectral information is obtained from every scan performed using routine protocols. Therefore, tissue characterization could be retrospectively performed on demand by measuring iodine density, effective atomic number, and ED.

ED generally corresponds to the physical density of the tissue. In clinical practice, ED has been used for optimizing the dose distribution in radiotherapy rather than for diagnostic purpose. Recently, Daoud et al. [14] have shown the potential benefit of ED in detecting lung lesions. ED images can improve the visualization of lung parenchymal lesions compared to conventional CT in patients with COVID-19 pneumonia [14]. In the present study, ED images derived from an unenhanced DECT clearly depicted PE, which could not be detected on conventional CT images. Due to the current pandemic situation, requests for non-contrast CT evaluations are increasing in patients with suspected COVID-19 [15]. In such patients, the pulmonary artery should also be carefully evaluated to detect potential complications, PE, particularly if the scan is performed using a dual-energy technique.

In summary, we report a case of PE detected on an unenhanced DECT using ED images. This shows the potential of DECT for patients who cannot use an iodinated contrast agent in CT scans due to poor renal function, allergy to iodinated contrast, or pregnancy.

## Data Availability

Data is contained within the article. No new data were created or analyzed in this study.
